# Sintering boron carbide ceramics without grain growth by plastic deformation as the dominant densification mechanism

**DOI:** 10.1038/srep15827

**Published:** 2015-10-27

**Authors:** Wei Ji, Sahibzada Shakir Rehman, Weimin Wang, Hao Wang, Yucheng Wang, Jinyong Zhang, Fan Zhang, Zhengyi Fu

**Affiliations:** 1 State Key Laboratory of Advanced Technology for Materials Synthesis and Processing, Wuhan University of Technology, Wuhan 430070, China

## Abstract

A new ceramic sintering approach employing plastic deformation as the dominant mechanism is proposed, at low temperature close to the onset point of grain growth and under high pressure. Based on this route, fully dense boron carbide without grain growth can be prepared at 1,675–1,700 °C and under pressure of (≥) 80 MPa in 5 minutes. The dense boron carbide shows excellent mechanical properties, including Vickers hardness of 37.8 GPa, flexural strength of 445.3 MPa and fracture toughness of 4.7 MPa•m^0.5^. Such a process should also facilitate the cost-effective preparation of other advanced ceramics for practical applications.

Traditionally, sintering is the densification process of compacted powder, in which interparticle pores are eliminated by atomic diffusion driven by thermal energy. The atomic diffusion may cause unavoidable grain growth besides contributing to densification, which are two competing processes in sintering^1^,^2^. For polycrystalline materials, higher density generally results in improvement of properties like strength and toughness, while the undesirable grain growth results in degradation of the same properties. Attempts have been made to maximize density while limiting the grains’ growth^3^,^4^,^5^,^6^. Chen and Wang proposed a two-step sintering method in which fully dense Y_2_O_3_ was prepared by a quick first-step heating at 1,250–1,350 °C to achieve an intermediate density (~80%) and then cooled down to a final-step holding at 1,150 °C for 20 h. The suppression of the final-stage grain growth is achieved and the finest grain size of fully dense ceramics is 60 nm, which is about 4–6 times the starting powder size due to the coarsening in the first-stage heating^3^. Kang and co-workers reported the sintering of 5 mol.% TiO_2_-excess BaTiO_3_ at relatively low temperature 1,290 °C with a very long soaking time, up to 100 h, and got pellets with relative density of 95% with limited grain growth^4^. Munir and Anselmi-Tamburini attempted to obtain nanometric cubic zirconia in a Spark Plasma Sintering (SPS) apparatus with a special die which could apply pressure up to 1,000 MPa. The temperature required to achieve the 95% relative density decreases with the applied pressure, which results in the grain size decreasing from 200 nm to 15 nm^5^. When sintering temperature is kept constant (1,200 °C), pressure influences the relative density, but has no effect on the grain size (about 80 nm)^6^.

Boron carbide (B_4_C) has attracted considerable attention because of its excellent properties, including high hardness (second only to diamond and cubic boron nitride), low density (the lightest ceramic), high melting point, good chemical stability and high neutron absorption cross section^7^. The combination of these properties makes B_4_C a fascinating material for various applications, such as light-weight armor, cutting tools, wear-resistant parts and neutron radiation absorbance in nuclear reactors^8^,^9^. However, the applications of monolithic B_4_C are limited by the difficulties in obtaining very dense bulk materials. Its low self-diffusion coefficient– resulting from strong covalent bonding between atoms, low plasticity, high resistance to grain boundary sliding and low surface tension– makes sintering the powder a difficult task^10^. Pressureless sintering of pure B_4_C is extremely difficult and obtains a relative density of 93% even at 2,375 °C^11^,^12^. Additives are helpful to the pressureless densification of B_4_C, but still temperatures as high as 2,150–2,250 °C are needed^13^,^14^,^15^. Pressure assisted sintering methods are encouraged and used in industry to prepare full density B_4_C with simple shapes. Pure B_4_C can be fully densified by Pressure assisted sintering at 2,050–2,200 °C and under applied loads of 30–40 MPa for 15–45 min^7^,^16^. Such conditions are economically too demanding in terms of energy consumption, so scientists are committed to find better process parameters. Ghosh *et al.* used a plasma pressure compaction technique (P^2^C) for densification of B_4_C particles with sizes around 800 nm at 1,750 °C, by external pressure of 88 MPa, and attained densities around 96–99% and grain sizes around 1.6–2.7 μm^17^. Li *et al.* densified nano-sized B_4_C particles (493 nm) with not high purity at 1,600 °C. But impurities composed of Cr, Mn, Fe, Ni, P, B and O have been found in the triple junction of the sintered samples, which contribute to densification by forming liquid phase. The low hardness (31 GPa) and high flexural strength (828 MPa) are more nearer to a B_4_C based cermet^18^. Moshtaghioun and co-workers analyzed the effect of SPS parameters, like temperatures and holding time, on the densification of B_4_C powders with average particle size around 500 nm. Fully dense B_4_C with mean grain size equal to 0.69 μm, 0.81 μm and 0.88 cm were consolidated under 75 MPa, at 1,700 °C for 3 min, 1,700 °C for 5 min and 1,750 °C for 3 min respectively^10^. Badica and co-workers suggested a high pressure (300 MPa) and low temperature (1,600 °C) SPS strategy and achieved dense B_4_C with relative density 95.6%. The fine structure (1.2–1.7 μm average grain size from 0.1–1 μm raw particle size) and residual B_2_O_3_ and carbon in the raw powder results in improvement of fracture toughness but decrease of Vickers hardness (K_1C_ = 6.6 MPa·m^1/2^, HV = 27.6 GPa) compared to corresponding values (K_1C_ = 3.8 MPa·m^1/2^, HV = 35.3 GPa) of the sample sintered by conventional SPS (2,100 °C, 50 MPa)^19^.

The effect of temperature on grain growth is thus: increasing temperature has almost no effect on grain size in the early stage, and then a rapid grain growth happens after an onset temperature T_g_ (Supplementary Fig. S1)^20^,^21^. By calculation of the grain-boundary mobility of polycrystalline, Holm and Foiles proved that there exists a characteristic temperature below which the smooth boundary from low mobility offers a mechanism for grain growth being stopped^22^. The effect of temperature on densification is a bit different: increasing temperature makes densification progress very slowly in the early stage, and then rapid densification happens after an onset temperature T_d_. Generally, T_d_ is lower than T_g_ for most materials (T_d_ < T_g_)^2^. So there exists a temperature region: T_d_–T_g_, in which ceramics can be densified with no or limited grain growth.

We propose to densify ceramic powders at a lower temperature, close to T_g_ in the T_d_–T_g_ region, to avoid the grain-boundary migration and grain growth, and at the same time, apply a relatively higher pressure to the compacted powder to attain full densification. If full densification can be achieved but with no grain growth, it is expected that the plastic deformation is the dominant mechanism, which is still a challenge to the traditional view and needs to be proven. We plan to sinter micro-size grain B_4_C, widely used in industry, by the new route in an SPS instrument and hope to provide an energy-efficient and high-performance technique for ceramics fabrication.

## Results

### Effects of processing parameters

**Figure 1:**
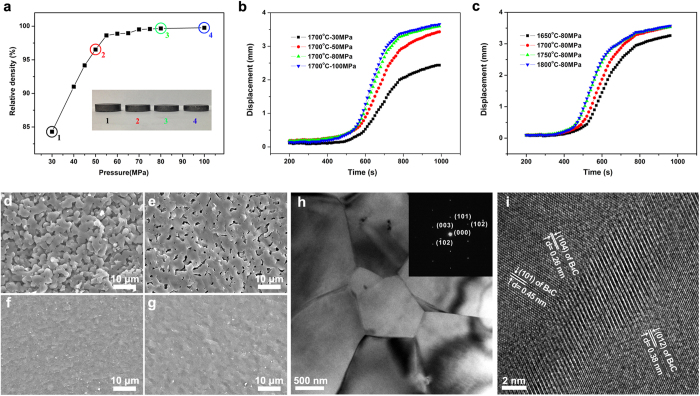
The effects of pressure and temperature on the densification of B_4_C powders. (**a**) The relative density as a function of pressure at 1,700 °C soaking for 5 min. (**b**) The sintering curves with different pressures at 1,700 °C. (**c**) The sintering curves with different temperatures under 80 MPa. (**d**–**g**) The fracture surfaces of sintered B_4_C with different pressures at 1,700 °C soaking for 5 min. (**d**) 30 MPa. (**e**) 50 MPa. (**f**) 80 MPa. (**g**) 100 MPa. (**h**,**i**) The detailed morphology characterization of a fully dense B_4_C fabricated under 1,700 °C, 80 MPa soaking for 5 min. (**h**) TEM bright field image with SAED patterns. (**i**) HRTEM image of the grain boundary with indexing of the lattice fringes along the two adjacent grains.

Figure 1 shows the effects of pressure and temperature on the densification process of B_4_C. The relative density increases sharply with the uniaxial pressure from 30 to 55 MPa, and then shows a slow rise up to 80 MPa, at the same temperature of 1,700 °C soaking for 5 min (Fig. 1a). The photographs inserted in Fig. 1a indicate that the thickness of samples changes markedly from 30 MPa to 50 MPa and slightly to 80 MPa. The displacement and time relationships, commonly known as the sintering curves, are shown in Fig. 1b,c. At a constant temperature of 1,700 °C, there is a small displacement under the pressure of 30 MPa, but the displacement increases by a factor of ~1.5 under the pressure of 80 MPa. Further increases of pressure, up to 100 MPa, have no obvious effect on the total displacement (Fig. 1b). Under a constant pressure of 80 MPa, the displacement at 1,650 °C is much less than that at higher temperatures. The total shrinkage exhibits similar values at the sintering temperatures from 1,700 °C to 1,800 °C (Fig. 1c). Figure 1d–g exhibit the fracture surfaces of B_4_C sintered under 30, 50, 80 and 100 MPa at 1,700 °C, respectively. Open pores exist in the sample under 30 MPa; only closed pores can be observed under 50 MPa. Fully dense bulks are fabricated with pressure bigger than 80 MPa.

The transmission electron microscopy (TEM) analysis in Fig. 1h,i shows detailed microstructure in the fully dense specimen. The bright field TEM image with selected area electron diffraction (SAED) patterns (Fig. 1h) exhibits a dense morphology of micro-sized grains with straight grain boundaries free of interphases. The high-resolution TEM (HRTEM) image (Fig. 1i) reveals distinct observation of a Moiré pattern (up to 10 nm wide) at the grain boundaries between the B_4_C grains. The Moiré pattern indicates that the grains are highly crystalline with clean grain boundaries in which obscured dislocations exist^23^. Indexing lattice fringes confirms the coincidence of (1 0 4) and (0 1 2) atomic planes along the two adjacent grains at the low-angle grain boundary (~2°) with lattice spacing of 0.26 and 0.38 nm, respectively. The unique morphology may be associated with plastic deformation under high contact pressure between grains. Such a coherent boundary suggests a strong bonding between B_4_C grains. It is well known that the grain boundary has a significant effect on the properties, especially the mechanical properties, of ceramics. Therefore it is predictable that good mechanical properties can be obtained for this fully dense B_4_C.

**Figure 2:**
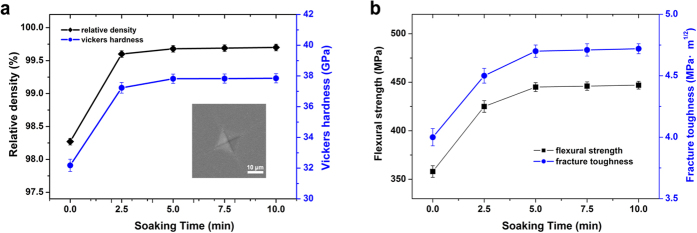
Effect of holding time on the densification and mechanical properties of sintered B_4_C at 1,700 °C and under 80 MPa. (**a**) Relative density and Vickers hardness. The inserted is a SEM image of indentation by Vickers hardness testing. (**b**) Flexural strength and fracture toughness.

Figure 2 shows the effect of holding time on the relative density and mechanical properties at 1,700 °C and under 80 MPa. The relative density and Vickers hardness increase rapidly with holding time and both reach high values in 5 min. Further increase of holding time has little effect on either relative density or Vickers hardness. The flexural strength and fracture toughness also increase rapidly with holding time and both reach relatively high values in 5 min. Further increase of holding time slightly increases the flexural strength and fracture toughness. B_4_C bulk sintered at 1,700 °C, under 80 MPa and with holding of 5 min obtained rather high relatively density (99.7%) and exhibits an attractive combination of mechanical properties, including Vickers hardness of 37.8 GPa, flexural strength of 445.3 MPa and fracture toughness of 4.7 MPa·m^0.5^, which is superior to the same polycrystalline materials reported, and close to the literature value of single-crystal B_4_C^16^,^17^,^18^,^19^,^24^,^25^. Longer soaking has no obvious effect on promoting the mechanical properties.

### Densification and grain growth

**Figure 3:**
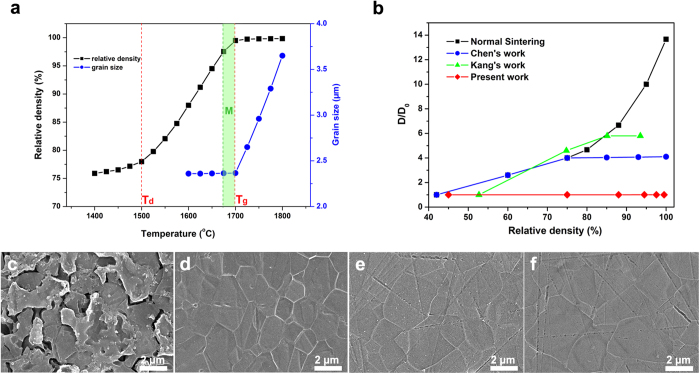
The details of change in relative density and grain size with changing temperature for B_4_C sintered under 80 MPa, held for 5 min. (**a**) Relative density and grain size of B_4_C as a function of temperature, sintered under 80 MPa, with soaking for 5 min. (**b**) Relationship between grain growth (D/D_o_) and relative density in different sintering methods. (**c**–**f**) Polished and etched surfaces of B_4_C sintered under 80 MPa, with soaking for 5 min, at 1,600 °C, 1,700 °C, 1,750 °C and 1,800 °C, respectively.

Figure 3 reveals the details of change in relative density and grain size with changing temperature for dense B_4_C sintered under 80 MPa, held for 5 min. As shown in Fig. 3a, the relative density increases dramatically with temperature from 1,500 °C, which indicates that the onset temperature for densification (T_d_) is about 1,500 °C. The high value of relative density (~95%) cannot be reached until 1,650 °C. The trend of grain growth differs from that of densification. The average grain size remains unchanged, as the raw particle size 2.36 μm until the temperature increases up to 1,700 °C, which indicates that the critical temperature for grain growth (T_g_) is about 1,700 °C, which is higher than that for densification. The polished and etched surfaces of B_4_C specimens sintered at different temperatures are shown in Fig. 3c–f. The grain sizes of B_4_C sintered at 1,600 °C, 1,700 °C, 1,750 °C and 1,800 °C are 2.36 μm, 2.36 μm, 2.96 μm and 3.65 μm, respectively. Therefore, there exists a useful temperature range “M” in T_d_–T_g_; the optimal range might be 1,675–1,700 °C for the micro-sized B_4_C in this work, in which high density can be achieved, but grains do not to grow (shown as the green range “M” in Fig. 3a). Chen’s idea is in fact to carry out the final-stage sintering in the T_d_–T_g_ region in 20 hours, which realizes densification, but keeps the grains from growing^3^. Kang’s attempt is sintering near the T_d_–T_g_ region to realize densification with limited grain growth, intentionally extending the holding time to 100 hours^4^. It is expected that densification will continue to develop with even longer soaking because of atomic diffusion, but will be very slow. Too long a soaking period in sintering is uneconomical. In fact, commonly, a sintering process is carried out in tens of minutes or several hours. In most cases, sintering temperatures are chosen bigger than T_g_, to attain high enough density in an acceptable time, which is why grain growth is inevitable^26^. It is worth pointing out that the temperature range “M” depends on the particles’ size (micro-size or nano-size) and the size distribution. Nano-sized particles with a big surface area are expected to complicate the region more, which needs to be studied in the future.

The relationship between grain growth (D/D_o_) and relative density for different sintering methods is summarized in Fig. 3b^3^,^4^. Compared with normal sintering, Kang’s low-temperature, long-soaking method and Chen’s two-step sintering, there is no grain growth in the densification process in the present work.

## Discussion

For pressure assisted sintering, the pressure is transmitted through the particles’ contacts. The contact force P can be expressed as^27^:


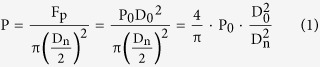


where D_n_ is the diameter of contact area, D_0_ is the particle diameter, P_0_ is the punch pressure.

In the early stage of sintering, the contact point is very small compared with the particle diameter, so the contact force can be rather high. As the punch pressure rises, the contact force increases, causing plastic yielding and an expansion of the points of contact into contact areas, which results in shrinkage or densification^28^. Our proposal is that if the pressure is high enough, which causes the contact area to be big enough, the plastic deformation can dominate densification.

At high sintering temperature, the yield stress for ceramics decreases. The dependence of yield stress σ_y_ on temperature for ceramics is given by^29^


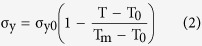


where σ_y0_ is a “pseudo” yield stress of fully dense ceramics at room temperature, and T_m_ is the melting point.

For B_4_C, extensive shock loading test has demonstrated that it is effective for protection against high-pressure impact. However, a dramatic decrease of anti-impact capability occurs at the pressure of 20 GPa^30^. The first-principles calculation also suggests that the destabilization pressure is between 18.9 ~ 22.8 GPa^31^. In addition, the critical pressure for the depressurization of B_4_C by amorphization is within the range of various reported values (10–15 GPa) of the loss in shear strength of B_4_C loaded above the Hugoniot elastic limit (HEL)^32^,^33^,^34^. It is well known that the yield stress under high speed impact is much higher than that under quasi-static pressing because of strain-rate hardening^35^,^36^,^37^. So the yield stress of fully dense B_4_C at room temperature σ_y0_ can be set as 10 GPa. From equation (2), the calculated yield stress σ_y_ for B_4_C at 1,700 °C is 2.79 GPa, which is the critical stress for plastic deformation. When the stress is higher than σ_y_, plastic deformation occurs.

**Figure 4:**
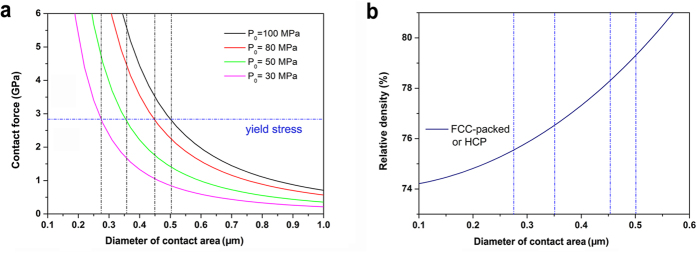
The calculated details of the contact area by plastic deformation under different punch pressures at 1,700 °C and the corresponding relative densities. (**a**) The relationship between the contact force and the contact area under different punch pressures from 30 to 100 MPa. (**b**) The relationship between relative density and the contact area for a packed grain structure.

Figure 4a shows the relationship between the contact force and the diameter of the contact area under different punch pressures. With a certain punch pressure, the contact force is very big at the beginning of pressing, and plastic deformation occurs. The contact force decreases with the increase of contact area. The plastic deformation will stop, while the contact force is lower than the critical yield stress σ_y_, which is what happens in the early stage of most pressure-assisted sintering when the pressure is not high enough. Increasing the punch pressure forms a wider contact area. At critical yield stress, the corresponding diameters of the contact areas, under punch pressures of 30 MPa, 50 MPa, 80 MPa and 100 MPa, are 0.28 μm, 0.36 μm, 0.45 μm and 0.50 μm, respectively, which shows a trend toward denser structure.

To relate the diameter of the contact area with relative density, Coble’s model of packed tetrakaidecahedral grains is widely accepted^38^. For fully dense structure in this work, hexagonal close-packed (HCP) or face-centered cubic (FCC) model is suitable to describe the well packed grains. The relationship is


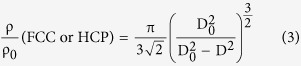


As seen in Fig. 4b, the corresponding relative densities for diameters of the contact areas 0.28 μm, 0.36 μm, 0.45 μm and 0.50 μm are 75.56%, 76.61%, 78.26% and 79.38%, respectively. The results indicate that a high relative density (~80%) can be achieved simply by plastic deformation under high pressure (≥ 80 MPa) at 1,700 °C. After this, the residue low porosity can be filled by pressure-assisted grain-boundary diffusion or Coble creep^21^,^39^. The schematic of the sintering route by plastic deformation as the dominant densification mechanism is shown in Supplementary Fig. S2. Maitre *et al.* found that high dislocation density occurs in ZrC grains sintered by high temperature (1,882–2,187 °C) and high applied load (100 MPa) during the final stage of the SPS process, and they proposed that a dislocation motion mechanism governs the densification^21^. In this present work, a high density of dislocations can also be found in the TEM micrographs performed by a two-beam experiment (Supplementary Fig. S3), which is in fair agreement with Maitre’s result in ZrC. In addition, recent literature has shown that dense dislocations play a vital role in both mechanical and functional properties of materials^23^. Whether there is contribution of dislocationson to the excellent performance of B_4_C will be an interesting area for future exploration.

In summary, we propose a simple method to sinter ceramic powders at a lower temperature close to the onset point of grain growth, and at the same time, to apply higher pressure to the compacted powder. Fully dense (99.7%) micro-sized B_4_C without grain growth was prepared at 1,700 °C and under pressure of (≥) 80 MPa in 5 min. The sample shows excellent mechanical properties, including Vickers hardness of 37.8 GPa, flexural strength of 445.3 MPa and fracture toughness of 4.7 MPa·m^0.5^. The plastic deformation under high pressure and at the chosen temperature is proven to be the dominant mechanism for the densification process.

## Methods

### Raw materials

Commercially available B_4_C powder (97% purity, Mudanjiang Diamond Boron Carbide Co., Ltd., China) was used as the starting material. According to the supplier’s data, the average grain size of the B_4_C powder is 2.36 μm. Detailed morphology analysis agrees well with the supplied data (Supplementary Fig. S4).

### Sintering of B_4_C

B_4_C was sintered from 2.5 g B_4_C powder by an SPS apparatus (Dr. Sinter-3.20MK II, Sumitomo Coal Mining Co. Ltd., Tokyo, Japan), using a cylindrical graphite die with inner diameter of 20 mm. The temperature was automatically raised by the SPS apparatus to 600 °C over a period of 3 min, and from this point and onwards it was monitored and regulated by an infrared thermometer focused on the surface of the die. A heating rate of about 100 °C/min was used. The compaction pressure was applied above 600 °C and maintained during the rest of the heating and soaking time in the SPS. Natural cooling began when the power was off at the end of the soaking, and the applied uniaxial pressure was removed at the same time. The selected ranges of pressure, holding temperature and time were 30–100 MPa, 1,600–1,800 °C and 0–10 min, respectively. All the factors were investigated using the controlling variate method: other parameters were fixed whenever the effect of one specific factor was studied. Hot pressing (HP) experiments of the same sample were used to eliminate the potential effect on the densification by electric in SPS. The results (Supplementary Fig. S5) have well confirmed the significant effect of high pressure and the plastic deformation as dominant densification mechanism with or without electricity.

### Microstructure and mechanical properties

The crystal structure of the as-preserved specimen was characterized by X-ray diffractometer (XRD, X’Pert PRO-PANalytical) with Cu Kα radiation. XRD analysis (Supplementary Fig. S6) indicates that both the raw powder and sintered bulk have highly crystallized rhombohedral structure (space group: R3^−^ m; 166), in good agreement with the standard PDF card (reported in JCPDS file No. 35–0798), and no secondary phase was detected. The relative densities of the sintered specimens were measured by “Archimedes” method with deionized water. The specimens’ surfaces were polished to 0.25 μm to get mirror-finish surfaces for successive microstructure examinations. The electrolytic etching was performed in 1% KOH solution with a current density of 0.1 A/cm^2^ for 20 ~ 30 s on the polished surfaces. Etched microstructures of the specimens were observed by scanning electron microscopy (SEM, Hitachi S3400). A thin foil of sintered material was prepared by mechanical thinning followed by ion milling and observed using transmission electron microscopy (TEM, JEOL JEM-2010HT), equipped with selected area electron diffraction (SAED) and high resolution transmission electron microscopy (HRTEM, JEOL JEM-2010FEF). The Vickers hardness was measured by a Vickers hardness tester (Wolpert-430SV) with applied load of 9.8 N for a dwell time of 15 s. The fracture toughness (K_1C_) of the ceramics was calculated after the indentation, using this equation^40^:


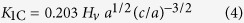


Here, H_v_ is the hardness, a is the impression radius, and c is the radial/median crack length. The flexural strength measurements were carried out via three point bending tests on 2 mm × 3 mm × 18 mm bars in a ceramic test system (MTS 810, MTS) with a span of 15 mm at a crosshead speed of 0.5 mm/min. The reported values of relative density and mechanical properties are the average of at least ten measurements.

## Additional Information

**How to cite this article**: Ji, W. *et al.* Sintering boron carbide ceramics without grain growth by plastic deformation as the dominant densification mechanism. *Sci. Rep.* **5**, 15827; doi: 10.1038/srep15827 (2015).

## Supplementary Material

Supplementary Information

## References

[b1] Kang S. J. L. Sintering: Densification, Grain Growth & Microstructure (Butterworth-Heinemann, 2005).

[b2] Guillaume B. G., Nathalie M. & Christian G. Sintering of ceramic powders: determination of the densification and grain growth mechanisms from the “grain size/relative density” trajectory. Scripta. Mater. 57, 137–140 (2007).

[b3] Chen I. W. & Wang X. H. Sintering dense nanocrystalline ceramics without final-stage grain growth. Nature. 404, 168–171 (2000). 10724165 10.1038/35004548

[b4] Lee M. G., Chung S. Y. & Kang S. J. L. Boundary faceting-dependent densification in a BaTiO_3_ model system. Acta. Mater. 59, 692–698 (2011).

[b5] Munir Z. A. & Tamburini U. A., The effect of electric field and pressure on the synthesis and consolidation of materials: A review of the spark plasma sintering method. J. Mater. Sci. 41, 763–777 (2006). 10.1007/s10853-020-05040-4 PMC7351546 32836384

[b6] Anselmi-Tamburini U. *et al.* Spark plasma sintering and characterization of bulk nanostructured fully stabilized zirconia: Part I. Densification studies. J. Mater. Sci. 19, 3255–3262 (2004).

[b7] Suri A. K., Subramanian C., Sonber J. K. & Murthy T. S. Synthesis and consolidation of boron carbide: a review. Int. Mater. Rev. 55, 4–39 (2010).

[b8] Thevenot F. Boron carbide-a comprehensive review. J. Eur. Ceram. Soc. 6, 205–225 (1990).

[b9] Domnich V., Reynaud S., Haber R. & Chhowalla A. M. Boron carbide: structure, properties, and stability under stress. J. Am. Ceram. Soc. 94, 3605–3628 (2011).

[b10] Moshtaghioun B. M. Effect of spark plasma sintering parameters on microstructure and room-temperature hardness and toughness of fine-grained boron carbide (B_4_C). J. Eur. Ceram. Soc. 33, 361–369 (2013).

[b11] Roy T. K., Subramanian C. & Suri A. K. Pressureless sintering of boron carbide. Ceram. Int. 32, 227–233 (2006).

[b12] Lee H. & Speyer R. F. Pressureless sintering of boron carbide. J. Am. Ceram. Soc. 86, 1468–1473 (2003).

[b13] Kim H. W., Koh Y. H. & Kim H. E. Densification and mechanical properties of B_4_C with Al_2_O_3_ as a sintering aid. J. Am. Ceram. Soc. 83, 2863–2865 (2000).

[b14] Ye F., Hou Z. P., Zhang H. J. & Liu L. M. Densification and mechanical properties of spark plasma sintered B_4_C with Si as a sintering aid. J. Am. Ceram. Soc. 93, 2956–2959 (2010).

[b15] Yamada S., Hirao K., Yamauchi Y. & Kanzaki S. Densification behaviour and mechanical properties of pressureless-sintered B_4_C-CrB_2_ ceramics. J. Mater. Sci. 37, 5007–5012 (2002).

[b16] Hayun S., Paris V., Driel M. P., Frage N. & Zaretzky E. Static and dynamic mechanical properties of boron carbide processed by spark plasma sintering. J. Eur. Ceram. Soc. 29, 3395–3400 (2009).

[b17] Ghosh D., Subhash G., Sudarshan T. S., Radhakrishnan R. & Gao X. L. Dynamic indentation response of fine-grained boron carbide. J. Am. Ceram. Soc. 90, 1850–1857 (2007).

[b18] Li X. G. *et al.* Densifi cation behavior and related phenomena of spark plasma sintered boron carbide. Ceram. Int. 40, 4359–4366 (2014)

[b19] Badica P. *et al.* Tough and dense boron carbide obtained by high-pressure (300 MPa) and low-temperature (1,600 °C) spark plasma sintering J. Ceram. Soc. Jpn. 122, 271–275 (2014).

[b20] Shi J. L. Relation between coarsening and densification in solid-state sintering of ceramics: Experimental test on superfine zirconia powder compacts. J. Mater. Res. 14, 1389–1397 (1999).

[b21] Gendre M., Maitre A. & Trolliard G. A study of the densification mechanisms during spark plasma sintering of zirconium (oxy-)carbide powders. Acta. Mater. 58, 2598–2609 (2010).

[b22] Holm E. A. & Foiles S. M. How grain growth stops: a mechanism for grain-growth stagnation in pure materials. Science. 328, 1138–1141 (2010). 20508126 10.1126/science.1187833

[b23] Kim S. I. *et al.* Dense dislocation arrays embedded in grain boundaries for high-performance bulk thermoelectric. Science. 348, 109–114 (2015). 25838382 10.1126/science.aaa4166

[b24] McMillan P. F. New materials from high-pressure experiments. Nat. Mater. 1, 19–25 (2002). 12618843 10.1038/nmat716

[b25] Domnich V., Gogotsi Y., Trenary M. & Tanaka T. Nanoindentation and Raman spectroscopy studies of boron carbide single crystals. Appl. Phys. Lett. 81, 3783–3785 (2002).

[b26] Kingery W. D. Densification during sintering in the presence of a liquid phase I. Theory. J. Appl. Phys. 30, 301–306 (1959).

[b27] Coble R. L. Diffusion models for hot pressing with surface energy and pressure effects as driving forces. J. Appl. Phys. 41, 4798–4807 (1970).

[b28] Arzt E., Ashby M. F. & Easterling K. E. Practical applications of hotisostatic pressing diagrams: four case studies. Metall. Trans. A. 14, 211–221 (1983).

[b29] Meyers M. A., Olevsky E. A., Ma J. & Jamet M. Combustion synthesis/densification of an Al_2_O_3_–TiB_2_ composite Mat. Sci. Eng A-Struct. 311, 83–99 (2001).

[b30] Chen M. W., McCauley J. W. & Hemker K. J. Shock-induced localized amorphization in boron carbide. Science. 299, 1563–1566 (2003). 12624264 10.1126/science.1080819

[b31] Yan X. Q. *et al.* Depressurization amorphization of single-crystal boron carbide. Phys. Rev. Lett. 102, 075505 (2009). 19257688 10.1103/PhysRevLett.102.075505

[b32] Zhang Y. *et al.* Shock compression behaviors of boron carbide (B_4_C) J. Appl. Phys. 100, 113536 (2006).

[b33] Vogler T. J., Reinhart W. D. & Chhabildas L. C. Dynamic behavior of boron carbide. J. Appl. Phys. 95, 4173–4183 (2004).

[b34] Ge D., Domnich V., Juliano T., Stach E. A. & Gogotsi Y. Structural damage in boron carbide under contact. Acta. Mater. 52, 3921–3927 (2004).

[b35] Ghosh A. K. Tensile instablility and necking in materials with strain hardending and strain-rate harding. Acta. Mater. 25, 1413–1424 (1977).

[b36] Wang Y. & Ma M. E. Strain hardening, strain rate sensitivity, and ductility of nanostructured metals. Mat. Sci. Eng. A-Struct. 375, 46–52 (2004).

[b37] Barsoum M. W., Zhen T., Kalidindi S. R., Radovic M. & Murugaiah A. Fully reversible, dislocation-based compressive deformation of Ti_3_SiC_2_ to 1 Gpa. Nat. Mater. 2, 107–111 (2003). 12612695 10.1038/nmat814

[b38] Coble R. L. Sintering crystalline solids. I. intermediate and final state diffusion models. J. Appl. Phys. 32, 787–792 (1961).

[b39] Coble R. L. A model for boundary diffusion controlled creep in polycrystalline materials. J. Appl. Phys. 34, 1679–1682 (1963).

[b40] Niihara K., Nakahira A. & Hirai T. The effect of stoichiometry on mechanical properties of boron carbide. J. Am. Ceram. Soc. 67, C13–C14 (1984).

