# Supplementary material for: Sintering boron carbide ceramics without grain growth by plastic deformation as the dominant densification mechanism
Source: Sci Rep. 2015 Oct 27;5:15827. doi: 10.1038/srep15827 (PMC4622079; doi:10.1038/srep15827)
Supplement: Supplementary Information [file srep15827-s1.pdf]

SUPPLEMENTARY INFORMATION

Sintering boron carbide ceramics without grain growth by plastic deformation as the dominant densification mechanism

Wei Ji [1,+], Sahibzada Shakir Rehman [1,+], Weimin Wang [1], Hao Wang [1], Yucheng Wang [1], Jinyong Zhang [1], Fan Zhang [1], Zhengyi Fu [1,*]

[1]State Key Laboratory of Advanced Technology for Materials Synthesis and Processing, Wuhan University of Technology, Wuhan 430070, China

[*]zyfu@whut.edu.cn

[+]these authors contributed equally to this work

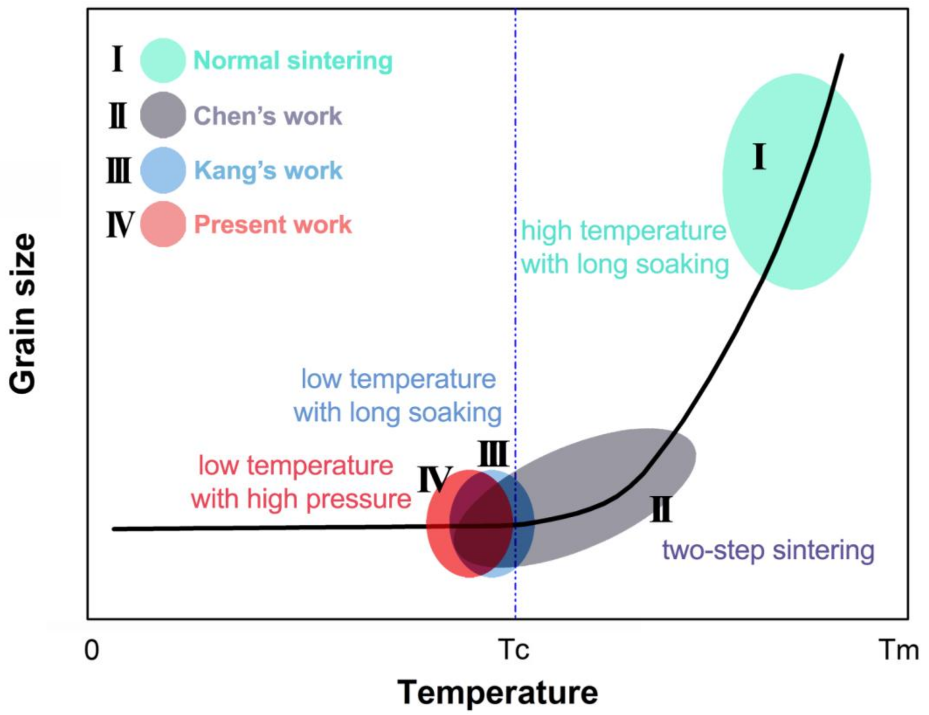

Supplementary Figure S1. Schematic diagram of ceramics' grain size by different sintering methods. I. normal sintering methods with high temperature[1], II. two-step sintering methods reported by Chen[2], III. low-temperature with long-soaking method proposed by Kang[3] and IV. low-temperature with high-pressure method in the present work.

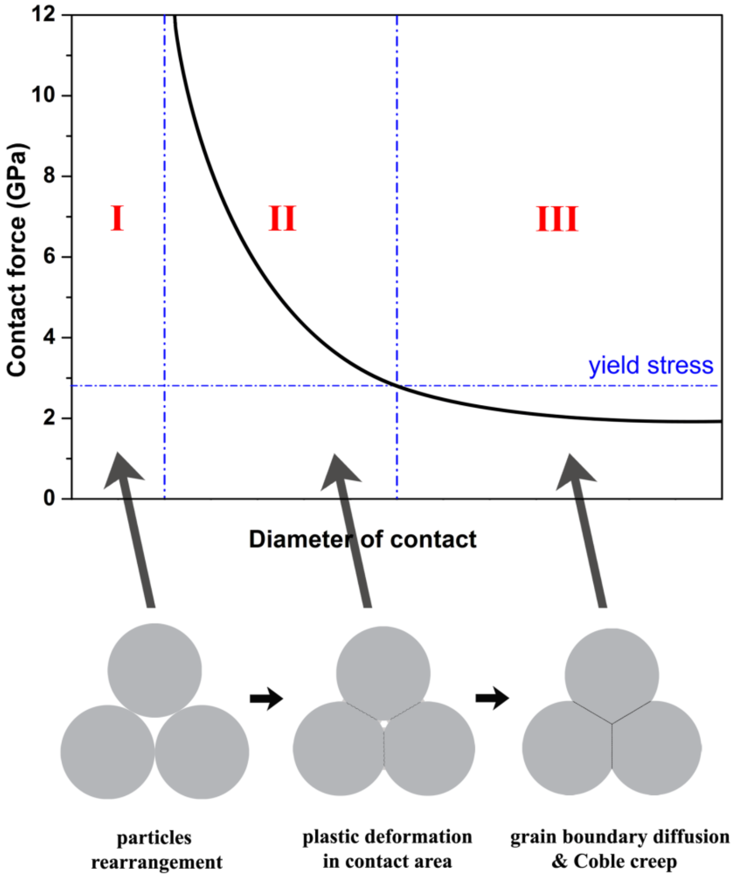

Supplementary Figure S2. Schematic of the sintering route by plastic deformation as the dominant densification mechanism. I. particles rearrangement in the green body, II. plastic deformation in contact area, III. grain boundary diffusion and Coble creep after plastic deformation[4].

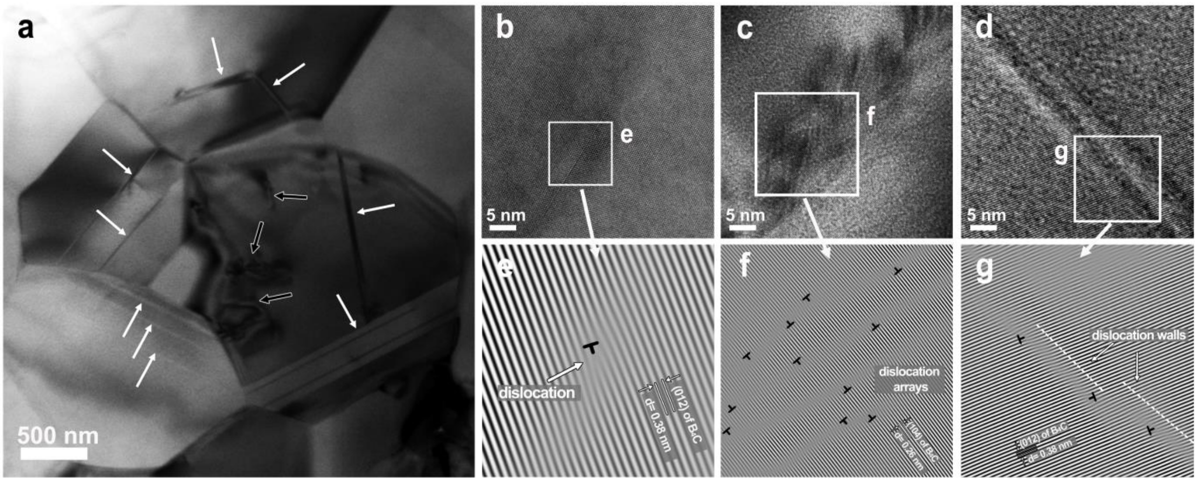

Supplementary Figure S3. TEM observations of B_4C sintered under 1,700 ℃, 80 MPa soaking for 5 min. (**a**) bright-field micrographs of the dense B_4C performed by two-beam experiments. The arrows show the presence of dislocations (black arrows), dislocation arrays and walls (white arrows), respectively. (**b**)-(**d**) HRTEM micrographs of dislocations, dislocation arrays and walls. (**e**)-(**g**) inverse fast Fourier transform (IFFT) images of the boxed regions.

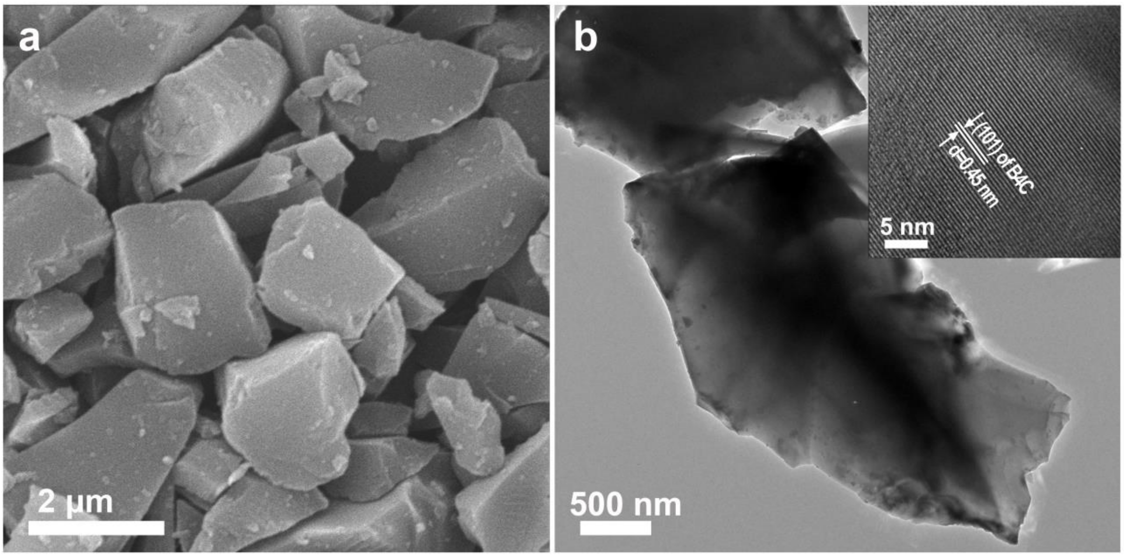

Supplementary Figure S4. Microstructures of the raw material. (**a**) the SEM image of B_4C powder. (**b**) TEM micrographs of B_4C particles. Inset, HETEM image of selected area.

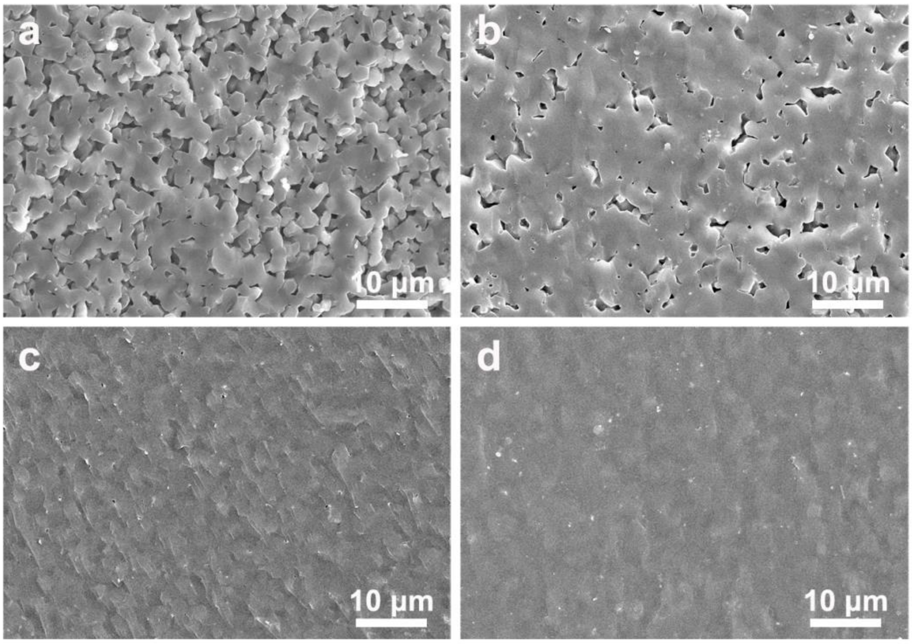

Supplementary Figure S5. The effects of pressure on the densification of B_4C by hot pressing. (a)-(d) The fracture surfaces of sintered B_4C with different pressures at 1,800 ℃. (a) 30 MPa. (b) 50 MPa. (c) 80 MPa. (d) 100 MPa.

The samples were sintered by hot pressing under pressures of 30-100 MPa at the temperature of 1,800 ℃ (recorded by an infrared thermometer focused on the surface of the graphite die in HP). Fig. S5 shows similar result to that we got by SPS (Fig.1d-g in the main manuscript). The results have well confirmed the significant effect of high pressure and the plastic deformation as dominant densification mechanism with or without electricity. We must point out that the recorded temperature difference between SPS and HP is just because of different heating methods: It is commonly observed that in SPS, the sample is exposed to a higher temperature than the one recorded and in the available literatures the temperature radial gradient is about 75 to 100 ℃[5,6]. On the contrary, in hot pressing the sample has a lower temperature than the recorded one as it is heated by thermal radiation from outside. Such temperature difference is acceptable.

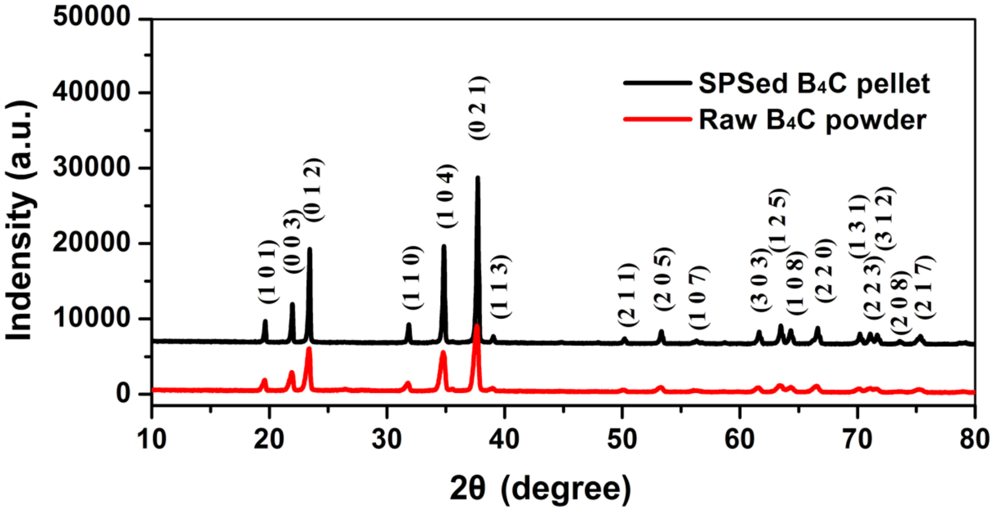

Supplementary Figure S6. X-ray diffraction spectrum of B_4C raw powder and bulks sintered under 1,700 ℃, 80 MPa soaking for 5 min. Both the raw powder and sintered bulk have highly crystallized rhombohedral structure (space group: $R\bar{3}m$; 166), in good agreement with the standard PDF card (reported in JCPDS file No. 35-0798), and no secondary phase was detected.

SUPPLEMENTARY REFERENCES

1. Kingery, W. D. Densification during sintering in the presence of a liquid phase. I. theory. *J. Appl. Phys.* **30**, 301-306 (1959).

2. Chen, I. W. & Wang, X. H. Sintering dense nanocrystalline ceramics without final-stage grain growth. *Nature*. **404**, 168-171 (2000).

3. Lee, M. G., Chung, S. Y. & Kang, S. J. L. Boundary faceting-dependent densification in a $BaTiO_3$ model system. *Acta. Mater.* **59**, 692-698 (2011).

4. Coble, R. L. A model for boundary diffusion controlled creep in polycrystalline materials. *J. Appl. Phys.* **34**, 1679-1682 (1963).

5. Shen, Z. J., Zhao, Z., Peng, H. & Nygren, M. Formation of tough interlocking microstructures in silicon nitride ceramics by dynamic ripening. *Nature*. **417**,266-269 (2002) ;

6. Antou, G., Mathieu. G., Trolliard. G., Maitre, A. Spark plasma sintering of zirconium carbide and oxycarbide: Finite element modeling of current density, temperature, and stress distributions. *J. Mater. Res.* **24**, 404-412 (2009)
